# Supplementary figures and images for: Meta-Analysis of the Impact of Far-Red Light on Vegetable Crop Growth and Quality
Source: Plants (Basel). 2024 Sep 6;13(17):2508. doi: 10.3390/plants13172508 (PMC11397353; doi:10.3390/plants13172508)

### Identification of studies via databases

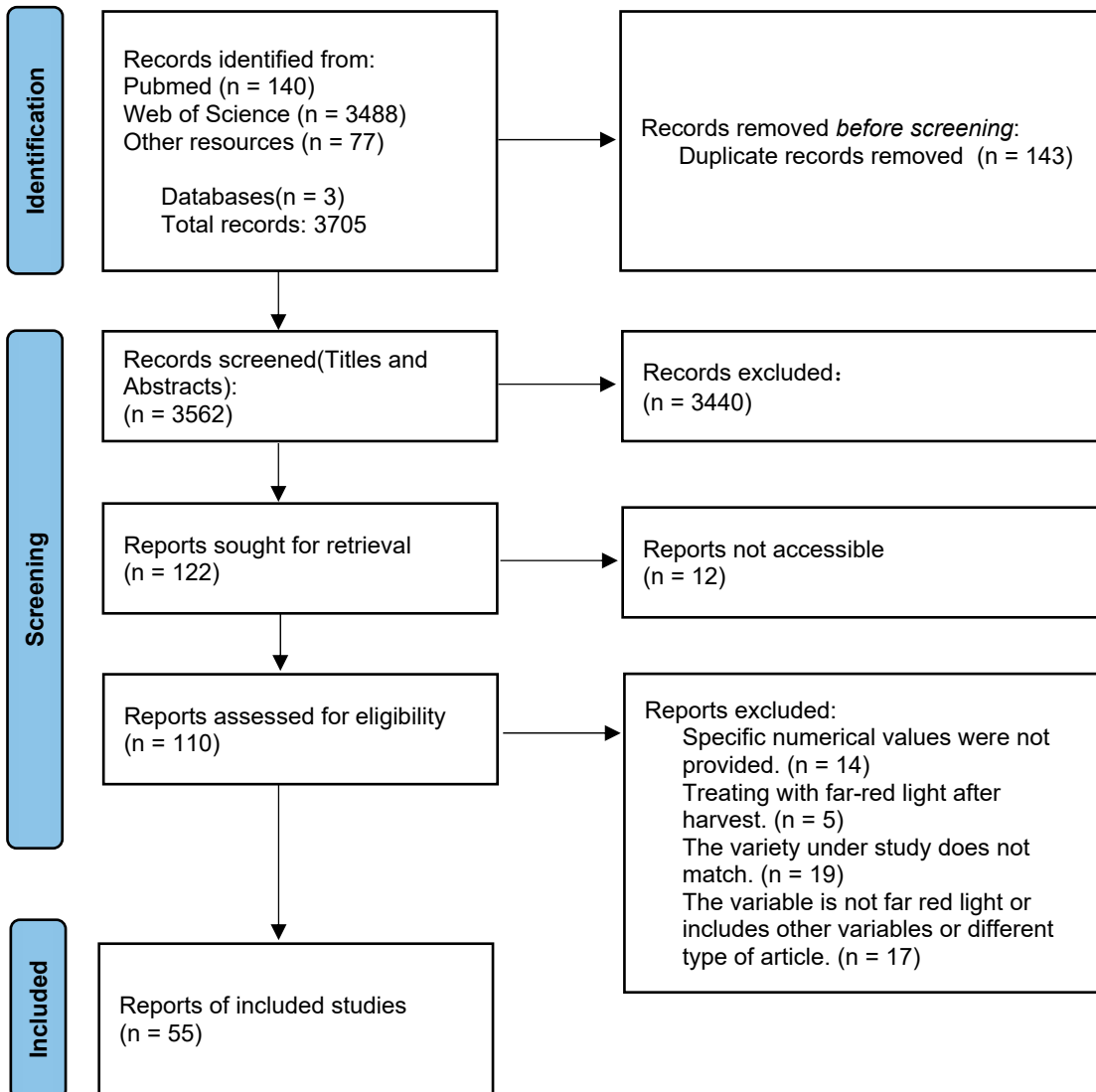

Supplement: Supplementary file 1 [file plants-13-02508-s001.zip › PRISMA_2020_flow_diagram_new_SRs_v1.pdf]
